# Predicting COVID-19 vaccination intentions: the roles of threat appraisal, coping appraisal, subjective norms, and negative affect

**DOI:** 10.1186/s12889-023-15169-x

**Published:** 2023-02-02

**Authors:** Xia Zou, Qiang Chen, Yangyi Zhang, Richard Evans

**Affiliations:** 1 School of Journalism and New Media, Xi’an Jiaotong University, Xi’an, China; 2 Faculty of Computer Science, Dalhousie University, Halifax, Canada

**Keywords:** COVID-19 vaccination, Subjective norms, Negative affect, Protection motivation theory

## Abstract

**Background:**

As a new disease, communities possess little natural immunity to COVID-19 and vaccines are considered critical to preventing and reducing the incidence of severe illness. This study, inspired by Protection Motivation Theory (PMT), examines the relationship between citizens’ threat appraisal, coping appraisal, subjective norms, negative affect, and their COVID-19 vaccination intentions.

**Methods:**

A sample of 340 citizens from two main cities in Mainland China, Xi’an and Wuxi, was used for data analysis. Structural Equation Modeling (SEM) was employed with latent and observed variables to test hypotheses. Data were analyzed using AMOS 24.0.

**Results:**

Several findings extend current understanding. Firstly, our proposed model explains 73% of the variance in vaccination intentions. Secondly, perceived severity only indirectly shapes COVID-19 vaccination intentions through negative affect. Thirdly, negative affect and response costs are negatively related to COVID-19 vaccination intentions. Finally, Perceived probability, subjective norms, response efficacy and self-efficacy are positively related to COVID-19 vaccination intentions; among them, self-efficacy contributes the most, followed by response efficacy and subjective norms, and lastly perceived probability.

**Conclusion:**

Theoretically, this study increases current understanding about subjective norms and affective responses. We provoke a certain amount of thought about the role of affect response in relation to threat appraisal and vaccination intentions. Specifically, governments must be vigilant that citizens’ negative affect, such as fear, may cause vaccine hesitation.

## Introduction

COVID-19 is an infectious disease caused by the SARS-CoV-2 virus. As of April 2022, the highly infectious disease has killed over 6.2 million people worldwide, creating enormous physical and psychological trauma to those affected [1]. Society has long been threatened by infectious diseases, such as SARS and bird flu, in addition to COVID-19, and the discovery of novel vaccines is considered an important measure to prevent continued infection [2]. As a new disease, communities possess little natural immunity to COVID-19; COVID-19 vaccines are, therefore, considered critical to preventing and reducing the incidence of severe illness [3]. Currently, although many countries are successfully managing infection rates and gradually re-opening borders to travelers, China is facing significant risk, and the speedy roll-out of COVID-19 vaccines to form an immunity barrier for citizens and communities.

Since 2020, China has made significant breakthroughs in the development of COVID-19 vaccines with 7 being released for emergency use. While vaccine development typically involves clinical trials requiring years or even decades of research to ensure good stability and preventive effect [4], COVID-19 vaccines have been developed in less than 3 years causing many citizens to question their effectiveness, side effects and preventive effects, leading to vaccine hesitancy [5, 6]. Recent data shows that the total number of fully vaccinated Chinese citizens is about 1.2 billion, accounting for 86.25% of the country’s population [7]; this means that there are still about 200 million people in China who remain un-vaccinated. At the same time, as new variants of the disease emerge, COVID-19 boosters are starting to be released [8]. As of April 2022, about 23.5% of Chinese citizens have received a COVID-19 booster leaving about 1.1 billion citizens still not receiving the booster [9]. Hence, it is crucial that we explore the determinants of COVID-19 vaccination intentions and propose targeted strategies to promote COVID-19 vaccination to avoid vaccine hesitancy.

Protection Motivation Theory suggests that personal information sources, threat appraisal, and coping appraisal, are correlated with protection motivation. It pays special attention to cognitive factors and provides a suitable model for research on protection intentions. Inspired by PMT, studies into COVID-19 vaccination intentions have mainly focused on investigating the influencing factors. For example, studies have identified that threat appraisal [10, 11], coping appraisal [12], personal information sources, such as the internet and interpersonal channels [13–15], maladaptive response reward [16], adaptive response [17], and rewards, including both external and internal rewards [18] correlate with citizens’ COVID-19 vaccination intentions. However, current research has insufficiently examined the associated social context and affective responses.

Social norms can encourage healthy behaviors, as well as unhealthy or harmful ones [1]. As is well-known, China has historically promoted collective consciousness [19]. Collectivism has developed into a cultural tradition and social norm in China, similar to rail transit and other infrastructure being spread throughout the socio-cultural psychological structure [20]. Chinese citizens rely on each other and try their best to obey the collective (i.e., group) to achieve development [20]. Obedience to the group and agreeance with common opinion often happens automatically with people tending to underestimate the extent to which their healthy behaviors and intentions are shaped by social norms [21]. In the context of COVID-19 vaccinations, it not only relates to its own health, but also the safety of others. From a collectivism perspective, the intentions of Chinese citizens to receive the COVID-19 vaccination may be affected by normative pressures; thus, further research is needed.

In addition to subjective norms, individuals’ COVID-19 vaccination intentions may be affected by negative affect [22]. Prior studies have found that more than 50% of people have felt worried or anxious during the pandemic [23]. However, studies that have drawn on PMT have suggested that negative affect, such as worry or anxiety, may occur, but does not necessarily affect protection motivation, while any fear caused by a threat is regarded as an irrelevant by-product [24]. The authors do not recognize the importance of affective responses to fear appeal [22]. Conversely, negative affect can help individuals think more clearly, allowing them to focus on the immediate situation, enabling quicker response speeds and decision making [25]. This means the relationship between negative affect and COVID-19 vaccination intentions remains understudied.

Accordingly, this study, inspired by the PMT model, aims to investigate the factors (i.e., threat appraisal, coping appraisal, subjective norm, and negative affect) that motivate the COVID-19 vaccination intentions of Chinese citizens.

### Theoretical framework

**Fig. 1:**
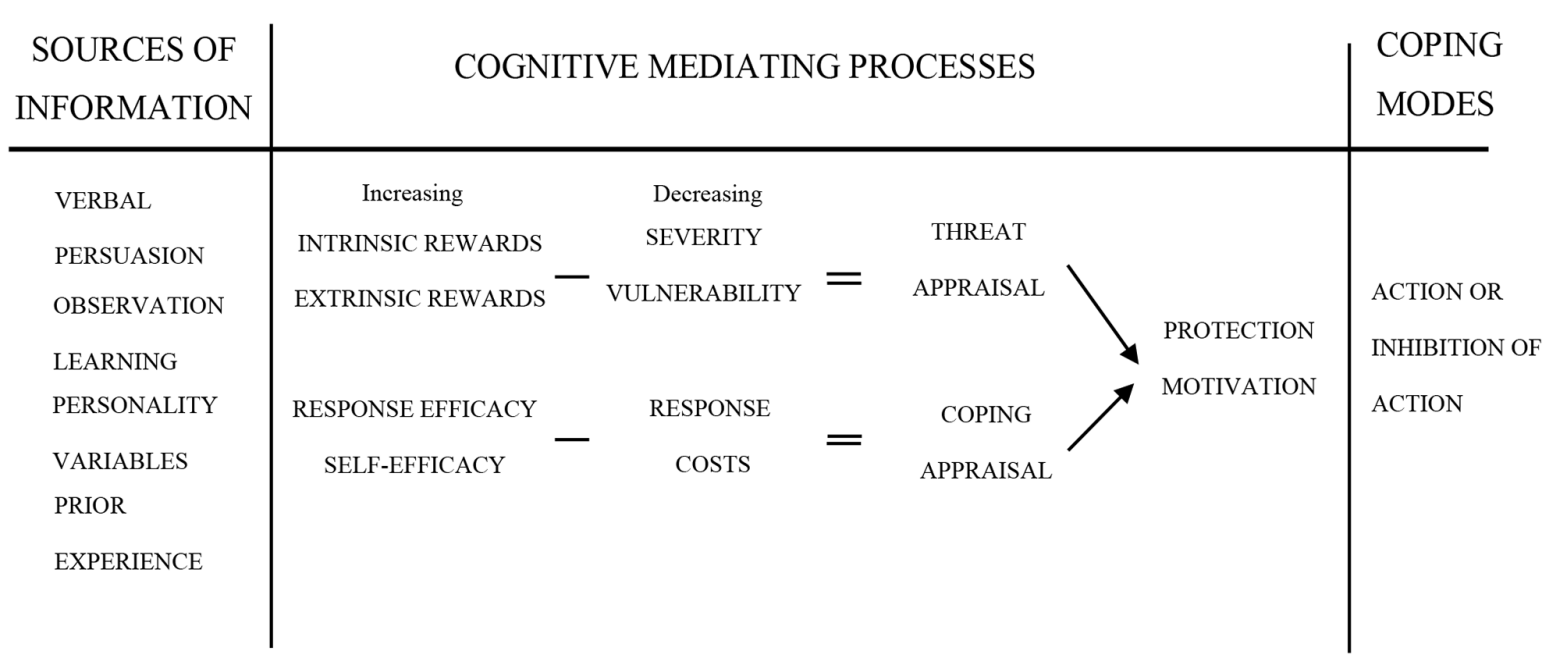
Protective Motivation Theory (Rogers, 1983)

Protection motivation theory, as illustrated in Fig. 1, was first proposed by Rogers [24]. The PMT model consists of three elements: sources of information, cognitive mediators, and coping models. Sources of information include verbal persuasion, observational learning, personality variables, and prior experience. Cognitive mediators include threat appraisal and coping appraisal. Threat appraisal refers to individuals’ perception of the risk [24] and includes perceived probability, perceived severity, intrinsic rewards, and extrinsic rewards. Coping appraisal refers to the perception of the effectiveness of protective measures and the ability for individuals to respond [24]. It consists of self-efficacy, response efficacy, and response costs. Coping models consist of action and inhibition of action. These elements share a certain relationship, that is, the sources of personal information are significantly related to threat appraisals and coping appraisals, while threat appraisals and coping appraisals are significantly related to protection motivation. Similarly, protection motivation significantly correlates with action or inhibition of action.

The PMT has been widely applied to studies on health protection intentions [26, 27] and is often applied when examining the prevention of infectious diseases and chronic non-communicable diseases, such as respiratory disease and cancers [28, 29]. At the same time, the theory has been applied to study citizens’ vaccination intentions. For example, the influenza vaccination [30], the Measles, Mumps, and Rubella (MMR) vaccination [31], the human papilloma vaccination [32], and the COVID-19 vaccination [16, 33].

Furthermore, the PMT model has been used extensively to study vaccination intentions, however, in its current state, the model lacks investigation of social context and affective response. Social context may affect individuals’ behaviors and intentions in a direct or indirect way, while investigation into personal social contexts can help explain people’s behavioral intentions [34]. Therefore, it is necessary to examine the relationship between subjective norms and COVID-19 vaccination intentions. Moreover, a fear appeal engages both cognitive and emotional processes [35], while negative affect, such as worry, is significantly related to individuals’ healthy behavior and intentions [36]. In summary, this paper aims to study the relationship among threat appraisal, coping appraisal, subjective norms, negative affect, and COVID-19 vaccination intentions.

### Hypotheses and concept model

#### Perceived severity, perceived probability, and negative affect

According to the PMT, threat appraisal includes perceived probability, perceived severity, intrinsic rewards, and extrinsic rewards. Intrinsic and extrinsic rewards refer to the perception of benefits derived from taking risky behaviors, often applicable to the study of non-benign behaviors, such as smoking [24]. The action of receiving the COVID-19 vaccination is a benign behavior, so we do not examine the variables of intrinsic and extrinsic rewards. This study only explores the relationship among perceived severity, perceived probability, and negative affect [12, 30].

Perceived probability refers to an individual’s cognitive judgment about the possibility of exposure to a risk. In this study, risk refers to the threat of contracting COVID-19. As a highly contagious infectious disease, COVID-19 has spread to almost every corner of the world. Extant research has found that individuals who assess their own exposure to more risk are prone to negative affect [37]. Negative affect refers to the negative emotional response about the given risk [38]. In this study, it refers to the negative affect of Chinese citizens when faced with COVID-19, such as fear or worry. In a study by Griffin et al. [38], the authors found that perceived probability is positively related to negative affect. Liao et al. [39] found that there is a significant positive correlation between perceived probability and anxiety in the context of the influenza disease. In addition, a study into adult twins in the United States revealed that the perception of COVID-19 exposure can trigger negative affect, such as fear [40].

Perceived severity refers to an individual’s cognitive judgment about the possible serious consequences of a risk. In this study, it refers to the perception of significant consequences from the risk of COVID-19 on the part of young Chinese citizens. High perceived severity can easily trigger anxiety about COVID-19 [41]. If someone is infected with COVID-19, they usually have a fever, a cough and so on. In severe cases, those infected can experience acute respiratory distress syndrome, septic shock, metabolic acidosis, coagulation dysfunction, and multiple organ dysfunction syndromes [42]. Recent data from China suggests that 25% of the population have experienced moderate to severe levels of stress or anxiety related symptoms in response to COVID-19 [43]. Studies have also shown that individuals who perceive serious consequences of contracting COVID-19 are more likely to have negative affect [11, 44]. Meanwhile, in a study by Lin and Bautista [45], they found that individuals’ cognition of the serious consequences caused by smog was positively correlated with negative affect (e.g., worry). Thus, we propose the following hypotheses:

H1: Perceived probability is positively related to negative affect.

H2: Perceived severity is positively related to negative affect.

### Negative affect and vaccination intentions

Affective responses play a central role in the study and practice of health protection intentions [46]. Individuals’ decisions and intentions are susceptible to affective responses [47]. Higher fear affects individuals’ mental health [48] and may shape their prevention intentions and behaviors towards COVID-19 [49]. Yang et al. [50] found that when individuals face the risk of COVID-19 infection, they are more likely to have negative affect, such as anxiety and sadness. Affective responses toward health problems directly affect peoples’ behavioral intentions to take protective measures [51, 52]. When people experience a life-threatening situation, negative affect may make them act in a certain way and, thus, individuals can benefit from negative affect when threatening situations are encountered [25]. During the COVID-19 pandemic, individuals are at risk of infection; subsequently, negative affect may prompt their COVID-19 vaccination intentions to lessen their chances of possible illness. Thus, we propose the following hypothesis:

H3: Negative affect is positively related to vaccination intentions.

### Perceived severity, perceived probability, and vaccination intentions

According to PMT, threat appraisal (i.e., perceived probability, perceived severity) is positively related to protective motivation. COVID-19 is a highly contagious disease that is mainly spread through air and close contact with droplets which cause infection [53]. Recent studies have found that people that perceive themselves as susceptible to COVID-19 are more likely to take the COVID-19 vaccination [54, 55]. In a survey of 547 US citizens by Ling et al. [30], they found that perceived probability was significantly positively correlated with COVID-19 vaccination intentions.

Individuals infected with COVID-19 generally present symptoms, such as a fever, cough, sore throat, loss of smell and taste, and body pain. Current research has discovered that COVID-19 also attacks individuals’ lungs [42], nervous systems [56], and other important human organs. The damage mechanism of COVID-19 is that protein S binds to the angiotensin-converting enzyme 2 receptor and invades alveolar epithelial cells, causing direct toxicity and excessive immune response [57]. Prior studies suggest that individuals who believe COVID-19 is associated with severe consequences are more likely to receive vaccination [30, 58]. Liu et al. [59] verified that, in the context of COVID-19, the perceived severity of migrant workers in Tianjin, Mainland China, was positively correlated with vaccination intentions. Thus, we propose the following hypotheses:

H4: Perceived probability is positively related to vaccination intentions.

H5: Perceived severity is positively related to vaccination intentions.

### Subjective norms and vaccination intentions

Subjective norms refer to the perceived social pressure to perform or not perform a behavior [34]. In this study, it refers to the perceived social pressure to be vaccinated against COVID-19. Subjective norms are one of the most important factors that correlate with individuals’ intentions [34]. The decision to receive a vaccination is often considered an individual or family decision and not a response to public health and the wider publicized benefits [60]. The positive attitude of others (e.g., close friends and family) to vaccination may effectively predict individuals’ vaccination intentions [61]. Previous studies have shown that subjective norm is positively related to vaccination intentions [1, 62, 63]. Thus, we propose the following hypothesis:

H6: Subjective norms are positively related to vaccination intentions.

### Self-efficacy, response efficacy, response costs, and vaccination intentions

According to PMT, self-efficacy is one of the main factors which affects coping appraisal. Self-efficacy refers to the subjective evaluation of one’s ability to successfully accomplish an activity [64]. In this study, it refers to the cognitive evaluation of one’s ability to achieve COVID-19 vaccination. Self-efficacy is often associated with vaccine availability, affordability, and accessibility, and is considered one of the major predictors of health-protective intentions [65]. Previous studies have found that self-efficacy is positively related to COVID-19 vaccination intentions [16, 66].

Response efficacy, which assesses the effectiveness of protective behavior in lessening the health threat, is one of the key predictors of vaccination intentions [66, 67]. In this study, it refers to individuals’ cognitive evaluation about the effectiveness of receiving the COVID-19 vaccination. Response efficacy usually reflects individuals’ perceptions of vaccine efficacy, that is, how effective is the vaccine in protecting them from the disease [68]. Studies have shown that personal cognition of vaccine prevention effect is positively related to vaccination intentions [30, 69].

In addition to self-efficacy and response efficacy, PMT suggests that coping appraisal also includes response costs. Response costs refer to the evaluation of the costs associated with the protective measures taken by individuals. In this study, it refers to individuals’ judgment about COVID-19 vaccine deficiency [70]. Lazarus and Folkman [71] proposed that cost judgements on protective behavior adoption affect people’s protective intentions and behaviors. The key barriers to receiving COVID-19 vaccination appear to be concerns about side effects and safety [72]. Studies have indicated that concerns about the side effects of vaccination significantly diminish the numbers of seasonal vaccinations [73]. Thus, we propose the following hypotheses:

H7: Response efficacy is positively related to vaccination intentions.

H8: Self-efficacy is positively related to vaccination intentions.

H9: Response costs are negatively related to vaccination intentions.

**Fig. 2:**
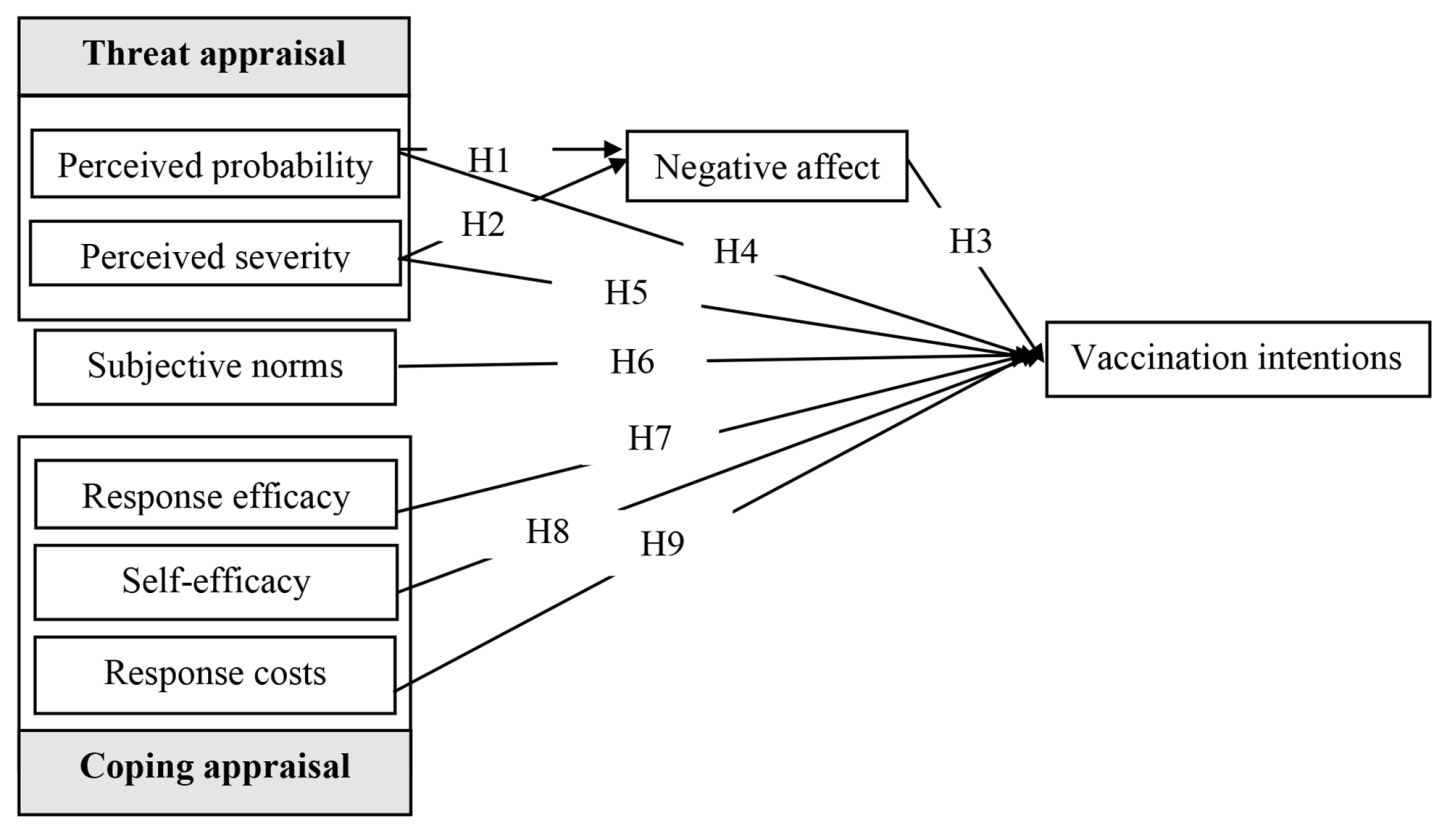
Proposed Conceptual Model

Based on our proposed hypotheses, Fig. 2 presents the conceptual model of this study.

### Methods

#### Participants and procedures

A pre-investigation was conducted in the cities of Wuxi and Xi’an, Mainland China. In total, 120 questionnaires were distributed with a valid callback rate of 80.83%. Following analysis, items with poor reliability and validity were eliminated. After removing the third item (Q3) of perceived severity (i.e., “if someone has COVID-19, the possibility of full recovery is low”), all latent variables’ value of Cronbach’s α and results from the Kaiser-Meyer-Olkin (KMO) test were greater than 0.7, indicating that the scale could be accepted.

The east city of Wuxi and the northwest city of Xi’an were chosen as survey sites due to their economies, population size, and differences in geographical position and COVID-19 risks. According to the seventh national census, Wuxi, a major city in Jiangsu province, has a permanent population of about 7.46 million. It has a well-established and diverse economy with industrial output mainly centered on its textile and precision machinery industries [74]. As an inland city, Wuxi is relatively safe with low COVID-19 risks. Xi’an is the largest city in Northwest China and has a permanent population of about 12.95 million. It has a well-established automotive manufacturing and aerospace industry. Due to its international airport, Xi’an faces significant risks of COVID-19 cases.

To conduct surveys in the two cities, four investigators were recruited through friends’ recommendations. Each city was assigned two investigators with each being paid about 300 yuan (approx. $47 USD). Before commencing data collection, we trained the two investigators online. Then, they were asked to collect questionnaires from March 1 to 28 March 2021. This period was selected due to it being in the early stages of the nationwide COVID-19 vaccination roll-out, and a substantial percentage of the population went back to school or returned to work after the Spring festival holiday. Thus, it was considered convenient for the collection of questionnaires. Our research adopted convenience and snowball sampling methods. Questionnaire responses were collected at universities, shops, businesses, and public institutions. The investigators approached potential participants, declared the intentions of the study, and then asked them to complete the questionnaire. All respondents were given 10 yuan (approx. $1.60 USD) for completing the questionnaire. In total, 372 questionnaires were received with 340 questionnaires (161 in Xi’an and 179 in Wuxi) meeting the requirements of this study. Table 1 presents the demographic information of the respondents who completed the questionnaire.

**Table 1:** Respondent Demographics (N = 340)

	Overall	Proportion (%)
Gender		
Female	166	48.8
Male	174	51.2
**Age**		
Below 20	75	22.1
20–30	115	33.8
31–40	123	36.2
41–50	16	4.7
Above 50	11	3.2
**Education**		
Less than high school	51	15
High school equivalent	140	41.2
Junior college and above	149	43.8
**Occupation**		
Student	117	34.5
Staff	178	52.4
Other	45	13.1
**Monthly Income**		
Less than ¥ 2,000	100	29.4
¥ 2,000 - ¥ 4,999	136	40
¥ 5,000 - ¥ 10,000	72	22.4
¥ 10,000 plus	32	8.2

This study was approved by the Institutional Review Board of Xi’an Jiaotong University. All methods used in the study were conducted according to the criteria set by the Ethics Committee of Xi’an Jiaotong University, while each participant reviewed and signed an informed consent form before participating in the study.

### Measures

*Perceived severity.* Perceived severity was measured using three items: (1) “If someone has COVID-19, the consequences can be terrible”, (2) “If someone has COVID-19, it can have a severe impact on their daily life”, and (3) “If someone has COVID-19, there will be serious health problems” (*M* = 4.37, *SD* = 0.75, Cronbach’s α = 0.85 ) [66]. A 5-point Likert-scale (1 = strongly disagree, 5 = strongly agree) was used to measure these three items.

*Perceived probability.* Perceived probability was measured using three items adapted from prior studies [75]. The sample items included: (1) “My circumstances make me vulnerable to contracting COVID-19”, (2) “It is possible for me to be infected with COVID-19 on the basis of my present situation”, and (3) “COVID-19 affects people my age and is highly infectious” (*M* = 2.73, *SD* = 0.95, Cronbach’s α = 0.82) A 5-point Likert-scale (1 = strongly disagree, 5 = strongly agree) was used to measure these three items.

*Self-efficacy.* Following the work of Mcmath and Prentice-Dunn [76], self-efficacy was measured using three 5-point Likert-scale items (1 = strongly disagree, 5 = strongly agree). These items included: (1) “It is easy for me to receive the COVID-19 vaccination”, (2) “Receiving the COVID-19 vaccination is a relatively simple process”, and (3) “I am not afraid of receiving the COVID-19 vaccine” (*M* = 3.89, *SD* = 0.88, Cronbach’s α = 0.92).

*Response efficacy.* Response efficacy was measured using three items adapted from prior studies [76]. The items included: (1) “Receiving the COVID-19 vaccine is an effective way to prevent COVID-19”, (2) “Receiving the COVID-19 vaccine protects me from contracting COVID-19”, and (3) “Taking the COVID-19 vaccine keeps me safe” (*M* = 4.10, *SD* = 0.80, Cronbach’s α = 0.93). These items were measured using a 5-point Likert-scale (1 = strongly disagree, 5 = strongly agree).

*Response costs.* Three 5-point Likert-scale items (1 = strongly disagree, 5 = strongly agree) were used to measure response costs. Following the work of Mcmath and Prentice-Dunn [76], the three items were: (1) “I think there will be unknown risks to my body if I receive the COVID-19 vaccine”, (2) “I think taking the COVID-19 vaccine will cause side effects”, and (3) “I think taking the COVID-19 vaccine may hurt my body” (*M* = 3.07, *SD* = 0.89, Cronbach’s α = 0.92).

*Negative affect.* To assess negative affect, respondents were asked: We would like to know your feelings about COVID-19. Please use a number from one to five, where one means you have none of the feeling and five means you have a lot of the feeling; When you think about COVID-19, how “worried”, “fearful”, and “sad” do you feel? (*M* = 3.14, *SD* = 0.98, Cronbach’s α = 0.89) [38, 77].

*Subjective norms.* Lu [77] suggested that three 5-point Likert-scale items (1 = strongly disagree, 5 = strongly agree) can be used to measure subjective norms. These items included: (1) “The people I value the opinions of support me in taking the COVID-19 vaccine”, (2) “The person whose opinion I value most wants me to take the COVID-19 vaccine”, and (3) “The organization that I study or work at wants me to take the COVID-19 vaccine” (*M* = 3.88, *SD* = 0.76, Cronbach’s α = 0.85).

*Vaccination intentions.* Vaccination intentions was measured using three 5-point Likert-scale items (1 = strongly disagree, 5 = strongly agree) [11]. These items included: (1) “I am willing to take the COVID-19 vaccine”, (2) “I plan on receiving the COVID-19 vaccination”, and (3) “I think I will definitely get vaccinated against COVID-19” (*M* = 3.88, *SD* = 0.93, Cronbach’s α = 0.95).

### Data analysis and hypotheses testing

#### Reliability and validity analysis

*Reliability analysis.* The reliability analysis was performed using SPSS 19.0 with results showing that the value of Cronbach’s α for perceived probability, perceived severity, self-efficacy, response efficacy, response costs, negative affect, subjective norms, and vaccination intentions ranged from 0.82 to 0.95, indicating good reliability.

*Convergent validity and discriminant validity analysis.* Confirmatory Factor Analysis (CFA) was conducted to test convergent validity and discriminant validity. Results show that the standard loading coefficient between observation items and corresponding latent variables were all greater than 0.50 (see Table 2). The value of Combined Reliability (CR) was greater than 0.70 [78], and the value of Average Variance Extracted (AVE) was greater than 0.50 [79]. Based on our analysis, it was concluded that all latent variables had good convergence validity. In addition, the square root of the AVE value was greater than the correlation coefficient between the corresponding latent variables and other latent variables, indicating that all latent variables had good discriminant validity [80].

**Table 2:** Results of confirmatory factor analysis

Variable	Item	Standard Load Coefficient	t Value	AVE	CR
Perceived probability	Q1	0.69	N/A	0.62	0.83
	Q2	0.90	12.89		
	Q4	0.76	12.43		
Perceived severity	Q5	0.86	N/A	0.67	0.86
	Q6	0.90	16.86		
	Q7	0.67	13.31		
Self-efficacy	Q8	0.82	N/A	0.79	0.92
	Q9	0.90	20.78		
	Q10	0.94	20.08		
Response efficacy	Q11	0.93	N/A	0.83	0.94
	Q12	0.94	33.69		
	Q13	0.87	25.26		
Response costs	Q14	0.90	N/A	0.79	0.92
	Q15	0.92	24.53		
	Q16	0.85	21.57		
Subjective norms	Q17	0.54	N/A	0.71	0.87
	Q18	0.95	11.18		
	Q19	0.96	11.20		
Negative affect	Q20	0.82	N/A	0.73	0.89
	Q21	0.96	19.83		
	Q22	0.79	16.98		
Vaccination intentions	Q23	0.92	N/A	0.85	0.94
	Q24	0.93	30.42		
	Q25	0.91	38.65		

*Note.* AVE = Average Variance Extracted; CR = Combined Reliability; N/A = Not Applicable

### Data strategy

Structural Equation Modeling (SEM) was employed with latent and observed variables to test the proposed hypotheses. Data were analyzed using AMOS 24.0. Table 3 presents the criterion for the fit index of the SEM model.

**Table 3:** Model fit indices

	Proposed model	Acceptable values
*χ* ^2^	434.022	/
*df*	228	/
*χ* ^*2*^ */df*	1.90	< 5 (Kline, 2005)
Goodness of Fit Index (GFI)	0.90	≥ 0.90 (Hair et al., 2006)
Adjusted Goodness of Fit Index (AGFI)	0.87	≥ 0.85 (Marsh et al., 1988)
Incremental Fit Index (IFI)	0.97	≥ 0.90 (Hair et al., 2006)
Root Mean Square Error of Approximation (RMSEA)	0.052 (90%CI = [0.044, 0.059])	< 0.08 (Hair et al., 2006)
Normed Fit Index (NFI)	0.94	≥ 0.90 (Hair et al., 2006)
Comparative Fit Index (CFI)	0.90	≥ 0.90 (Bentler, 199)
Standardized Root Mean Square Residual (SRMR)	0.05	< 0.10 (Kline, 2005)

*Note.* CI = Confidence Interval

**Fig. 3:**
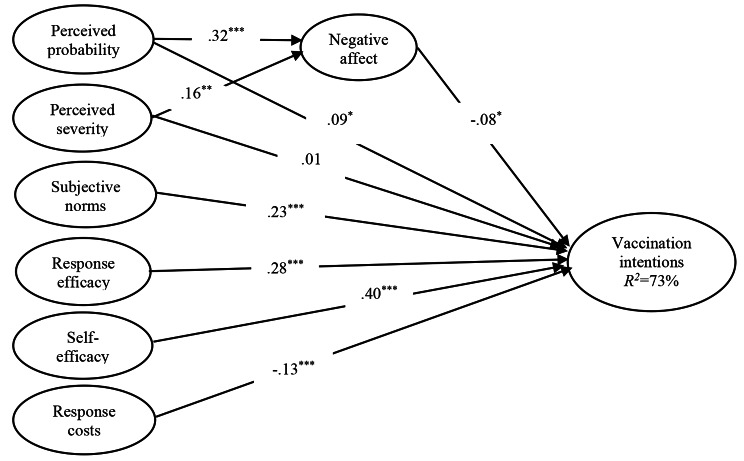
Results of the proposed conceptual model with statistically significant and unsignificant paths. Standardized solution is reported. Significance key: *p < .05, **p < .01, ***p < .001

The measurement model was estimated and demonstrated a good fit: *χ2* (228) = 434.022, *χ2*/*df =* 1.90, *p* < .001, RMSEA = 0.052, SRMR = 0.05. The value of GFI, CFI, IFI and NFI were all greater than or equal to 0.90, and the value of AGFI was greater than 0.85 (see Table 3). These results suggest that the model is acceptable. Figure 3 presents the standardized path coefficients in the obtained model.

### Hypotheses testing

The results of this study, as shown in see Fig. 3, reveal that perceived probability (β = 0.32, *p* < .001) and perceived severity (β = 0.16, *p* < .01) are positively related to negative affect. Thus, H1 and H2 were supported. Negative affect (β = − 0.08, *p* < .05) is negatively related to vaccination intentions. Thus, H3 was unsupported. The relationship between perceived severity (β = 0.01, *p* > .05) and vaccination intentions was unsignificant. Thus, H5 was unsupported. Perceived probability (β = 0.09, *p* < .05), subjective norms (β = 0.23, *p* < .001), response efficacy (β = 0.28, *p* < .001), and self-efficacy (β = 0.40, *p* < .001), are positively related to vaccination intentions, meaning that H4, H6, H7 and H8 were supported. Response costs (β = − 0.13, *p* < .001) are negatively related to vaccination intentions. Thus, H9 was supported.

As shown in Fig. 3, the value of R^2^ is 73%, indicating that the proposed model explains 73% of the variance in vaccination intentions.

## Discussion

### Summary of findings

This paper, inspired by the PMT model, studied citizens from Xi’an and Wuxi, Mainland China, with the aim of identifying the relationship among threat appraisal, coping appraisal, subjective norms, negative affect, and COVID-19 vaccination intentions. The study reveals that perceived severity and perceived probability are positively associated with negative affect. This finding suggests that a high perception of possible serious consequences from COVID-19 and a high self-assessment of the possibility of exposure to COVID-19 motivate individuals’ negative affect. Similarly, it can be found that Chinese citizens pay greater attention to their risk exposure, and higher risk exposures are more likely to trigger negative affect.

In addition, pandemic-induced anxiety, loss, and mental fatigue affect individuals’ health behaviors and vaccination intentions [81]. Our results reveal that negative affect is negatively correlated with COVID-19 vaccination intentions, which is different to findings from existing studies [45]. Negative affect has been used effectively for a long time in behavioral intentions change studies [82]. For example, negative affect helps to improve individuals’ intentions to quit smoking [83], and fear has been found to help people drive safely [84], while the relationship between negative affect and COVID-19 vaccination intentions is complicated. During the COVID-19 pandemic, complexity is further exacerbated by the emotional nature of individuals, coupled with anti-vaccine sentiment [85]. Previous studies have confirmed that under uncertain and uncontrollable situations, people focus on reducing negative affect, rather than reducing potential threats via behavioral changes [86]. Therefore, encouraging vaccination intentions with an individual’s negative affect may instead stimulate further fear and an inability to engage in preventative measures (e.g., vaccination) [85]. A study of Chinese adults during the COVID-19 pandemic illustrated that exposure to images of COVID-19 heightened anxiety and led to vaccine hesitancy [87]. In a community where emotions are generally high, these negative affects must be treated with caution rather than inadvertently heightening them in ways that would be counterproductive [85]. At present, to improve the implementation of China’s epidemic control policies, media outlets are continuously strengthening personal perceptions of COVID-19 risks. It is necessary to be vigilant against the excessive negative affect, which may cause vaccine hesitancy.

Our results reveal that the relationship between perceived severity and vaccination intentions is not significant, which is inconsistent with previous studies [11]. This may be due to the relatively safe living environments experienced in Xi’an and Wuxi. By the end of the current investigation, large-scale infection had not occurred in the two cities and most citizens’ knowledge about the consequences of COVID-19 was limited to indirect experience. Similarly, constant dilution of COVID-19 and sporadic deaths etc. suggest that the potentially severe consequences of COVID-19 may be overestimated. Hence, personal perceptions of the severity of COVID-19 do not significantly increase vaccination intentions. Perceived probability directly shaped vaccination intentions, which is consistent with previous findings [88]. That is, if individuals perceive themselves as vulnerable to COVID-19, they may actively seek vaccination. Thus, advising citizens about COVID-19 being a highly contagious disease that can spread rapidly through droplets, contact and aerosol etc. may help increase citizens’ vaccination intentions.

Self-efficacy plays the most important role in promoting vaccination intentions. Self-efficacy strongly promotes personal efforts to achieve healthy behaviors and intentions [65]. Since COVID-19 vaccines were authorized for emergency use, the Chinese government has provided many convenient channels for promoting COVID-19 vaccination, such as providing door-to-door services, giving one set of incentives and so on. These measures have proved beneficial to individuals’ self-efficacy and have improved COVID-19 vaccination intentions. However, in light of more than 200 million Chinese citizens still not receiving the vaccination, the Chinese government should try to provide more vaccine options (such as the Pfizer vaccines, etc.). These options shall contribute to enhancing citizens’ self-efficacy and may enhance Chinese citizens’ vaccination intentions.

Subjective norms are positively associated with vaccination intentions. This may be due to China’s unique cultural context and epidemic control strategies. The Chinese government advocates strict epidemic prevention and control policies. Once citizens are infected with COVID-19, governments close buildings or even cities. Under the collectivist culture, when an individual contracts COVID-19 without being vaccinated, they are more likely to be isolated and punished. During the COVID-19 pandemic, most citizens chose to receive the COVID-19 vaccine to avoid punishment or some type of loss.

Response efficacy is positively associated with vaccination intentions. In general, the better individuals perceive the COVID-19 vaccine prevention effect, the stronger their willingness to be vaccinated against COVID-19 is. As there are still 200 million citizens in China who have not received the COVID-19 vaccination, they may doubt the efficacy of the vaccine, leading to vaccine hesitancy. In future, it is necessary to strengthen the research and development of COVID-19 vaccines, improve the preventive effects of vaccines, respond to individuals’ concerns about preventive effects, and popularize knowledge about vaccines’ protective effects. These measures can be conducive to the promotion of China’s COVID-19 vaccines, including boosters.

Response costs are negatively correlated with vaccination intentions. China’s COVID-19 vaccines had a relatively short development time; besides, the epidemic situation in China is mainly sporadic and thus, lacks the environment for large-scale clinical trials. This tends to exacerbate individuals’ fear and hesitation about the side effects of COVID-19 vaccines. In future, strengthening international cooperation to develop more advanced COVID-19 vaccines, thereby reducing side effects, may help to increase the COVID-19 vaccination intentions of citizens. Meanwhile, Chinese officials should disclose more objective side effects and actively respond to citizens’ concerns to avoid vaccine hesitancy.

### Theoretical and practical contributions

The theoretical value of this study is that we have innovatively increased the investigation into subjective norms and affective responses. First, collectivism has a long history in China. Collectivism traditions shape Chinese citizens’ vaccination intentions through normative pressures. This research examined meso-level situational factors (subjective norms), which is a useful supplement and attempt to the PMT model. Second, this study examines the relationship between negative affect and individuals’ COVID-19 vaccination intentions. The results reveal that negative affect is negatively related to individuals’ vaccination intentions, which provides an innovative framework for studying Chinese citizens’ COVID-19 vaccination intentions.

The practical significance of this study is the provision of certain references for effectively promoting COVID-19 vaccination intentions. Through analysis of 340 samples from two main cities in China, we concluded that, with a tradition of collectivism and consciousness, subjective norms play an important role in promoting COVID-19 vaccination intentions. In addition, self-efficacy, response efficacy, response costs, and perceived probability, play a significant role. Moreover, this study provokes a certain amount of thought about the role of affect response in relation to threat appraisal and vaccination intentions. We found that negative affect is negatively correlated with vaccination intentions. This means that if the Chinese government wants to improve citizens’ willingness to receive the COVID-19 vaccination, it must be vigilant that negative affect, such as fear, may cause vaccine hesitation.

### Limitations and future research

Two limitations exist in our study. First, we analyzed only cross-sectional data from a one-month period in 2021 and did not undertake a longitudinal study. Second, some possible factors (e.g., information sources) may have been ignored, which requires further research. Accordingly, future studies should explore the following key aspects: (1) conducting a longitudinal comparative analysis; and (2) exploring the relationship between personal information sources on COVID-19 vaccination intentions, etc.

## Data Availability

All the data reported or analyzed in this paper is available by e-mail from the corresponding author, Qiang Chen.
